# CD19^+^CD24^hi^CD38^hi^ regulatory B cells deficiency revealed severity and poor prognosis in patients with sepsis

**DOI:** 10.1186/s12865-022-00528-x

**Published:** 2022-11-10

**Authors:** Chunmei Wang, Huihui Xu, Rui Gao, Fengying Leng, Fangjie Huo, Yinzhen Li, Siting Liu, Mingzheng Xu, Jianwen Bai

**Affiliations:** 1 Department of Emergency Medicine and Critical Care, Shanghai East Hospital, Nanjing Medical University, Nanjing, 211166 Jiangsu Province China; 2 Department of Emergency Medicine and Critical Care, Shanghai East Hospital, Tongji University School of Medicine, 150 Jimo Road, Shanghai, 200120 China; 3 Key Laboratory of Molecular Virology and Immunology, Institut Pasteur of Shanghai, Chinese Academy of Sciences, Shanghai, China; 4 University of Chinese Academy of Sciences, Beijing, China; 5 Department of Respiratory and Critical Care Medicine, Affiliated Hospital of Jining Medical University, Jining, 272067 Shandong Province China; 6 Department of Respiratory Medicine, Xi’an No. 4 Hospital, Xi’an, 710004 Shanxi Province China; 7 Medical School, Tongji University, Shanghai, 200120 China

**Keywords:** Sepsis, Flow cytometry, B-lymphocytes, Interleukin-10, Regulatory B cells, Prognosis

## Abstract

**Background:**

Sepsis still remains a major challenge in intensive care medicine with unacceptably high mortality among patients with septic shock. Due to current limitations of human CD19^+^CD24^hi^CD38^hi^ Breg cells (Bregs) studies among sepsis, here, we tried to evaluate Bregs in severity and prognostic value in patients with sepsis.

**Methods:**

Peripheral blood from 58 patients with sepsis and 22 healthy controls was analyzed using flow cytometry to evaluate the frequency and number of Bregs. All cases were divided into non-survived or survived group after 28 days followed up. Spearman's correlation analysis was performed on Bregs frequency and clinical indices. The area under the curve was acquired using the receiver operating characteristic analysis to assess the sensitivity and specificity of Bregs for outcome of sepsis. Survival curve analysis and binary logistic regression were applied to estimate the value of Bregs in prognosis among cases with sepsis.

**Results:**

Sepsis patients had decreased proportions and number of Bregs. Sepsis patients with low frequency of Bregs were associated with an increased risk of septic shock. Bregs frequency is inversely associated with lactate, SOFA, and APACHE II and positively correlated with Tregs frequency. Low levels of Bregs closely correlated with septic outcomes. Numbers of Bregs were prediction factors for poor prognosis.

**Conclusions:**

Frequency and number of Bregs decreased, and Bregs deficiency revealed poor prognosis in patients with sepsis.

**Supplementary Information:**

The online version contains supplementary material available at 10.1186/s12865-022-00528-x.

## Introduction

Sepsis is defined clinically as the presence of acute syndrome of infection with the development of new organ dysfunction, an increase of 2 points in the Sequential (Sepsis Related) Organ Failure Assessment (SOFA) [1]. Sepsis remains one of the major challenges in intensive care medicine, its incidence increasing continuously over the past decades [2, 3]. Despite massive efforts in sepsis research, in-hospital mortality (20.3%) is broadly more common among patients with hospital-onset sepsis than those (18.3%) with community-onset sepsis [4, 5], mortality in patients with septic shock still remains unacceptably high [3]. Therefore, emphasis has been focused on identifying patients who are at a higher risk of developing septic shock, early treatment, and early prognosis assessment. Research in sepsis has shown that the pathology of sepsis results not only from the presence of an infection, but also from the dysregulated host response to an infection [6]. Recent accumulating evidence depicts that sepsis is characterized by an initial overwhelming production of proinflammatory cytokines followed by immunosuppression, with increase of the regulatory T cells (Tregs) population [7–9]. Interleukin-10–producing B cells are a newly described subset of B cells with regulatory function. Mizoguchi and collaborators identified regulatory B cells (Bregs) as an IL-10–producing B-cell subpopulation and introduced the term “regulatory B cells [10].” In humans, Blair and coworkers described Bregs as CD19^+^CD^24hi^CD^38hi^, a phenotype that generally defines human transitional B cells [11, 12]. Since these seminal observations, a considerable body of evidence of CD19^+^CD24^hi^CD38^hi^ has been shown to prevent tissue injury, autoimmunity, chronic graft-versus-host disease (cGVHD), infection, and cancer [13–17]. So far, Bregs in sepsis, however, remain incompletely understood.

An increased frequency of circulatory Tregs in patients diagnosed with septic shock is significantly associated with immunosuppression, meanwhile percentage of Tregs increased as early as 3 days after shock, but the absolute number still was lower than in healthy donors [18]. Recent study supports Bregs, like Tregs, capable of negatively modulating T-cell–dependent autoimmune responses in an Ag-specific manner [19–21]. However, the function and clinical relevance of Bregs during sepsis remains unclear and little is presently known about the survival of patients following diagnosis. The prime aim of this exploratory study was to investigate Bregs in septic shock development and prognosis using a whole blood staining approach by flow cytometry. Our study demonstrated that sepsis patients with high frequency of Bregs had a lower risk of septic shock development. Our results showed that low levels of Bregs were associated with an increased risk of non-survival in patients with sepsis. This association might be due to decreased IL-10-producing B cells, and elevated IL-10-producing T cells. Findings indicated that Bregs might be applied to identify sepsis patients who are at risk of septic shock development and evaluate outcome during sepsis state.

## Materials and methods

### Subjects

**Fig. 1:**
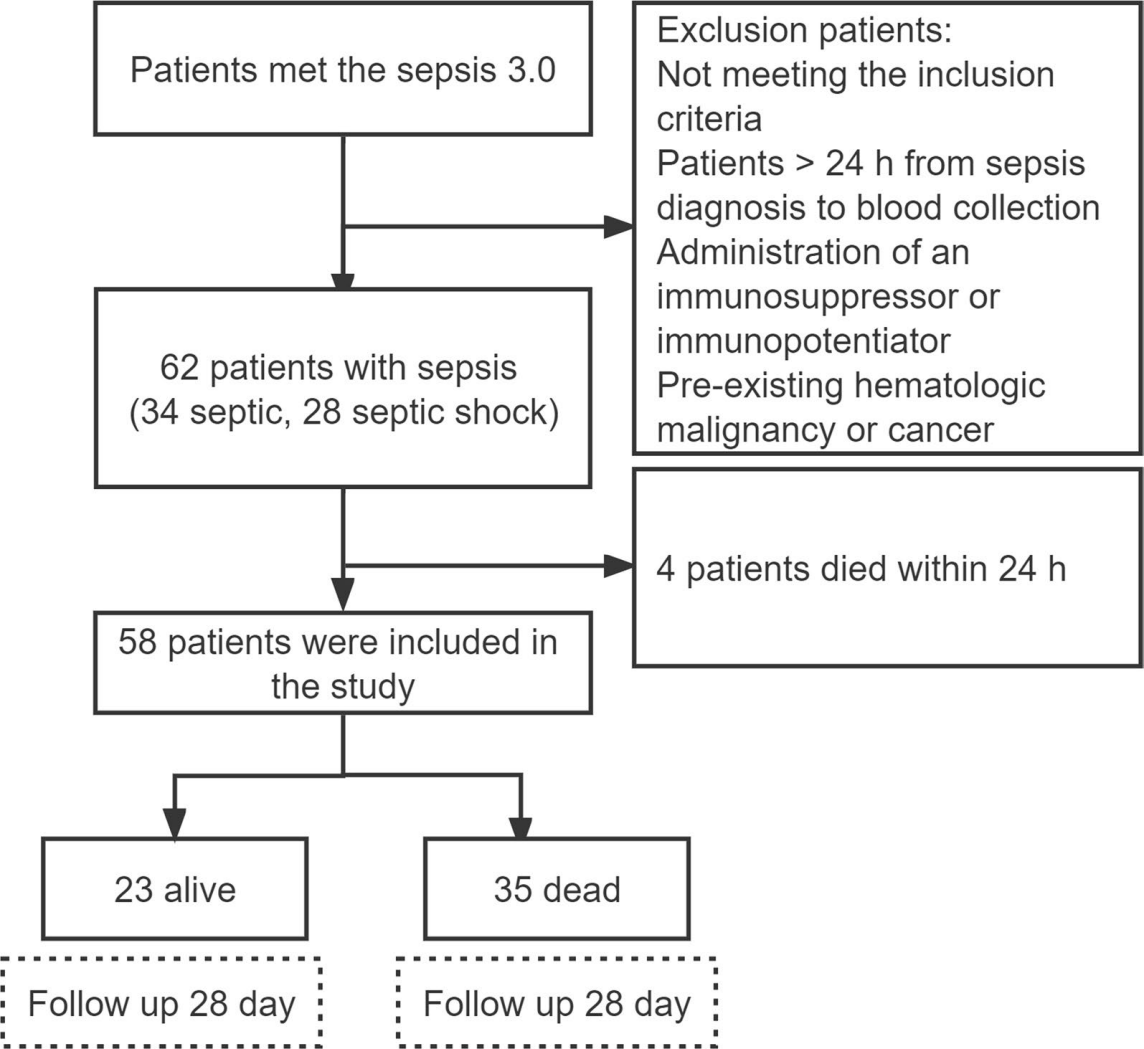
Flow chart for sepsis patients

Sepsis inclusion criteria were based on the Surviving Sepsis Campaign definition 3.0 [1]. Overall, 58 septic patients, diagnosed with sepsis (n = 26) and septic shock (n = 32) at intensive care unit (ICU) of Shanghai East Hospital between May 2016 and May 2018 were enrolled into study (Fig. 1). Healthy controls inclusion criteria were as follows: Matched age to the sepsis group of patients; good health; no history of chronic or metabolic diseases (e.g., hypertension, diabetes, coronary heart disease, hepatitis, and hyperthyroidism); and not using antibiotics within 3 days before enrollment. Healthy control group exclusion criteria were the same as those for sepsis.

The study was approved by the Institutional Ethics Authorities (No. 2015-028, Tongji University, China). All enrolled patients and healthy controls signed informed consent forms.

Eight mL of peripheral venous blood samples collected from septic patients at day 1 after admission and from healthy controls were subjected to lymphocyte separation within 3 h and flow cytometry analysis. The following general clinical data were collected and recorded at day 1: demographic characteristics (age and sex), main diagnosis, vasopressor use, ICU days, mechanical ventilation, infection site, and pathogenic bacterial agent [based on qualitative culture or metagenomic next generation sequencing (mNGS)] (Table 1). The white blood cell (WBC), creatinine, bilirubin, C-reactive protein (CRP), procalcitonin (PCT), blood lactate (Lac), Acute Physiology and Chronic Health Evaluation II (APACHE II) score, and Sequential Organ Failure Assessment (SOFA) score of patients on the day of admission were recorded (Additional file 1: Table S1). Twenty eight-day follow-up was complete. All patients with sepsis were divided into survivors (Alive) and non-survivors (Dead).

**Table 1:** Clinical data in elderly patients with sepsis and healthy donors

	Healthy	Sepsis	t/F	*p*
n	22	58		
Age (year)	75.2 ± 7.0	78.6 ± 8.1	1.832	0.0707
Male gender, n (%)	10 (45.5)	38 (65.5)	0.167	0.1281
Septic shock, n (%)		26 (44.8%)		
Mechanical ventilation, n (%)		6 (10.3%)		
Infection site, n(%)		Lung, 38 (65.5)		
		Biliary tract 9 (15.5)		
		Digestive tract 5 (8.6)		
		Urinary tract 3 (5.2)		
		Skin/ soft tissue 3 (5.2)		
Etiology, n (%)		Bacterial, 38 (65.5)		
		Fungal, 9 (15.5)		
		Viral, 11 (19.0)		

### CD19^+^CD24^hi^CD38^hi^ Bregs analysis by flow cytometry

Peripheral blood mononuclear cells (PBMCs) were isolated from blood samples using standard Ficoll gradient centrifugation. The isolated PBMCs were stained at room temperature for 15 min with antibodies against CD45 (OKT3), CD3 (OKT3), CD19 (SJ25C1), CD20 (2H7), CD24 (ML5) and CD38 (HIT2). Cells were gated on CD19^+^ B cells and CD19^−^ fraction after gating out CD3^+^ T cells. B cells were selected on a biparametric CD3^−^CD19^+^ dot-plot and Bregs were selected on CD24^hi^ CD38^hi^ circled from the CD19^+^ gate.

300 μL fresh whole blood was stained with antibodies against CD45 (OKT3) and CD33 (WM53) after lysing red blood cells. Then we selected WBC as CD45^+^ cells based on a biparametric CD45/SSC dot plot. By gating out CD33^lo^SSC^hi^ polymorphonuclear (PMN), PBMCs were selected and the percentage of PBMCs in CD45^+^ cells was calculated. Absolute numbers of CD45^+^ lymphocytes per microliter were then calculated. Next, B cells were selected based on a biparametric CD19/SSC dot plot and the percentage of CD19^+^ B cells in PBMCs was calculated. Then, Bregs were gated based on CD3^−^CD19^+^CD24^hi^CD38^hi^ and the percentage of Bregs in CD19^+^ cells was calculated. Meanwhile, the appropriate irrelevant anti-mouse isotype controls IgG1-FITC, APC and PE were used.

The absolute numbers of Bregs were calculated according to standard flow cytometry criteria for lymphocyte subset identification. The absolute number of CD19^+^ B lymphocytes was calculated by determining the percentage of CD19^+^ B cells in peripheral blood lymphocytes multiplied by the total number of lymphocytes per microliter measured using URIT-2900. The absolute number of CD19^+^CD24^hi^CD38^hi^ was calculated by multiplying the total number of B lymphocytes calculated by the percentage of positive cells in CD19^+^ B cells. Absolute numbers were expressed as cells per milliliter.

### Type 1 T helper (Th1) and Tregs analysis by flow cytometry

PBMCs were obtained from blood sample cells at day 1 after patients admission. The isolated PBMCs were stained for 15 min at room temperature with anti-CD3-BUV395, CD4-BV510, CD25-PE-Dazzle594, CD127-BV650, CXCR3-PECy7 and CCR6-BV785. PBMCs were suspended in staining buffer and detected by FACS Fortessa flow cytometry. T cells were selected on a biparametric CD3/SSC dot plot. Tregs were selected on CD4^+^CD25^+^CD127^+^ and Th1 were selected on CD4^+^CXCR3^+^CCR6^**−**^.

### IL-10^+^ Breg cells analysis by flow cytometry

PBMCs were first stained with combinations of cell surface markers, including anti-human CD3, CD19, CD20 monoclonal antibody (BD) for 30 min at 4 ˚C, and then stimulated with 50 ng/mL phorbol 12-myristate 13-acetate (PMA) (Sigma), 1 μg/mL ionomycin (Sigma), and 5 μg/mL brefeldin A (Biolegend) for 5 h at 37˚C. Finally, cells were washed twice and stained with zombie yellow™ dye (Biolegend) to eliminate dead cells. Unstimulated cells were used as controls for the following analyses. Intracellular cytokine staining was then carried out using anti-human interleukin-10 (IL-10) monoclonal antibodies (BD) after permeabilization and fixation. Labeled cells were acquired on LSRFortessa flow cytometer (BD Biosciences), and data were processed using Flowjo software (Tree Star).

### ELISA for plasma cytokine

A total of 150 μL plasma was collected after centrifugation on day 1 after diagnosis and then stored at −80 ℃ for further detection. Human plasma IL-10 was detected by ELISA kit (R&D Systems) per manufacturers instructions.

### Statistical analysis

The clinical data were first tested for normality and homogeneity of variance. Data conforming to a normal distribution and homogeneity of variance were expressed as the mean ± SD, and the t-test or analysis of variance was used for statistical analysis. Data were shown as the mean ± SD. The χ2 test was used to compare the enumeration data. Mann–Whitney U-test was used for the comparison of the two groups. One-way analysis of variance (ANOVA) was for comparison among multiple groups, followed by Kruskal–Wallis test with Dunns multiple comparison test when the values were not normally distributed. Correlation analysis was analyzed using Pearson’s chi-square test. Statistical tests were performed using SPSS 20.0 (SPSS, Chicago, IL, USA) and GraphPad Prism 8.0 (GraphPad Software, San Diego, CA, USA). *p* < 0.05 was considered statistically significant.

## Results

### Frequency of Bregs decreased in patients with sepsis

**Fig. 2:**
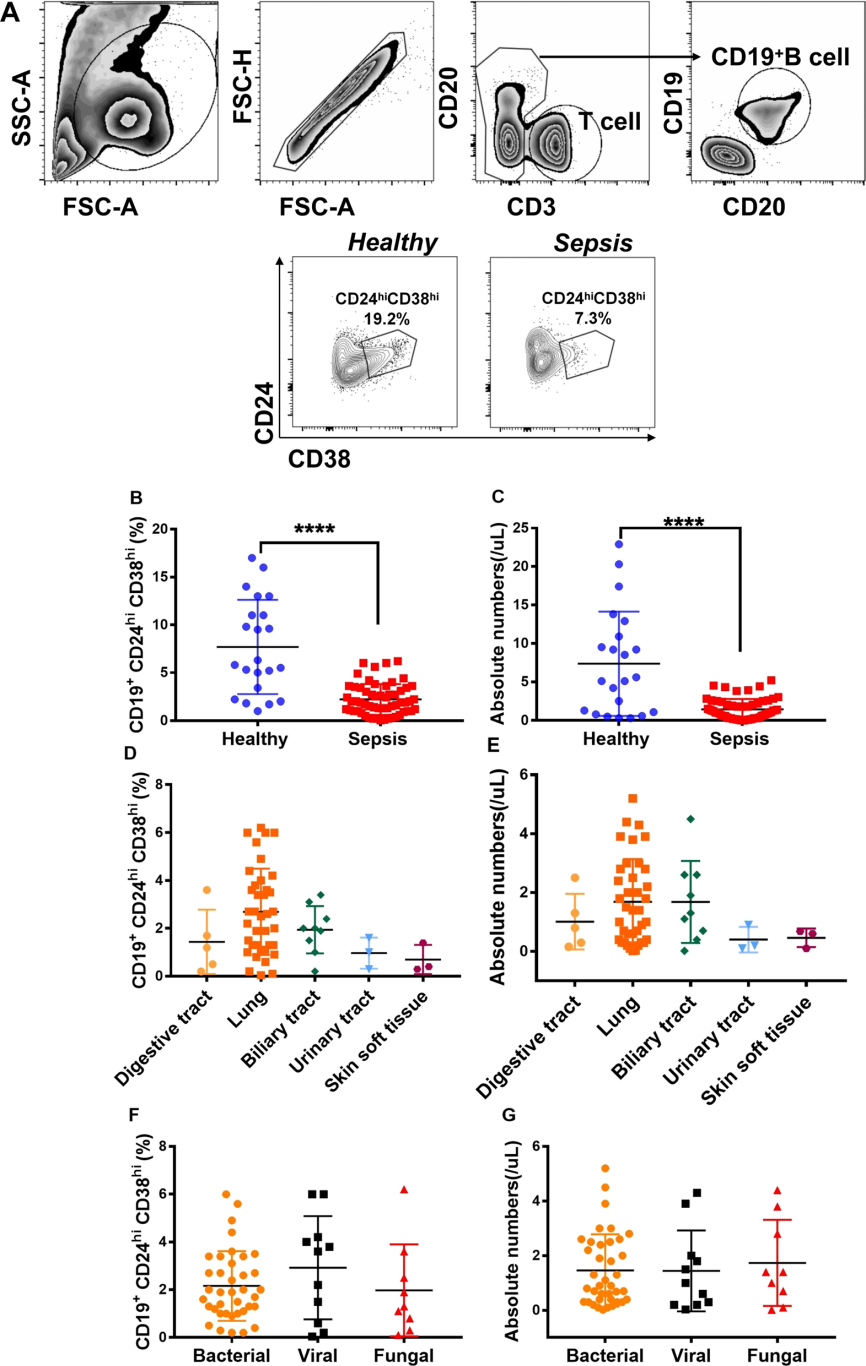
Bregs were decreased in sepsis patients on day 1. **A** Gating strategy of Bregs in representative healthy and sepsis patients. > 1 million events in the lymphocyte gate were collected. **B** Bregs requency in total B cells and **C** absolute number per microlitre of blood of Bregs in all healthy and sepsis subjects. Absolute number of Bregs per microlitre of blood was calculated by multiplying the total number of B lymphocytes calculated by the percentage of positive cells in Bregs in lymphocytes. **D** Frequency and **E** absolute number of Bregs in sepsis patients caused by digestive tract, lung, biliary tract, urinary tract, and skin/soft tissue infection. **F** Frequency and **G** absolute number of Bregs in sepsis patients with bacterial, viral, and fungal causes. Two-tailed t-test with unequal variance or Kruskal–Wallis test. ****p < 0.0001

Blood samples were collected at day 1 after patients were admitted in ICU and prepared within 3 h for flow cytometry after collection. The frequency of classical human Bregs was distinguished by CD3^−^CD19^+^CD24^hi^CD38^hi^ (Fig. 2A). 58 septic patients (38 males, 20 females) and 22 healthy controls were recruited for the study (Table 1, Additional file 1: Table S1). There was no significant age or sex differences between healthy controls and septic ones (Table 1). Infection sites were defined as lung, biliary tract, urinary tract, digestive tract, and skin/soft tissue. Among the patients with sepsis, pneumonia represented the initiation of infection (38/58); bacterial infection was the main cause of sepsis (38/58) (Table 1). We found that the frequency and absolute number of Bregs were both significantly decreased in septic patients compared with healthy controls (Fig. 2B–C). Frequency and absolute number of Bregs did not differ significantly among infection sites (Fig. 2D–E). No significant differences were observed for the etiology of sepsis (Fig. 2F–G).

### Low frequencies of Bregs were associated with development of septic shock in patients with sepsis

**Fig. 3:**
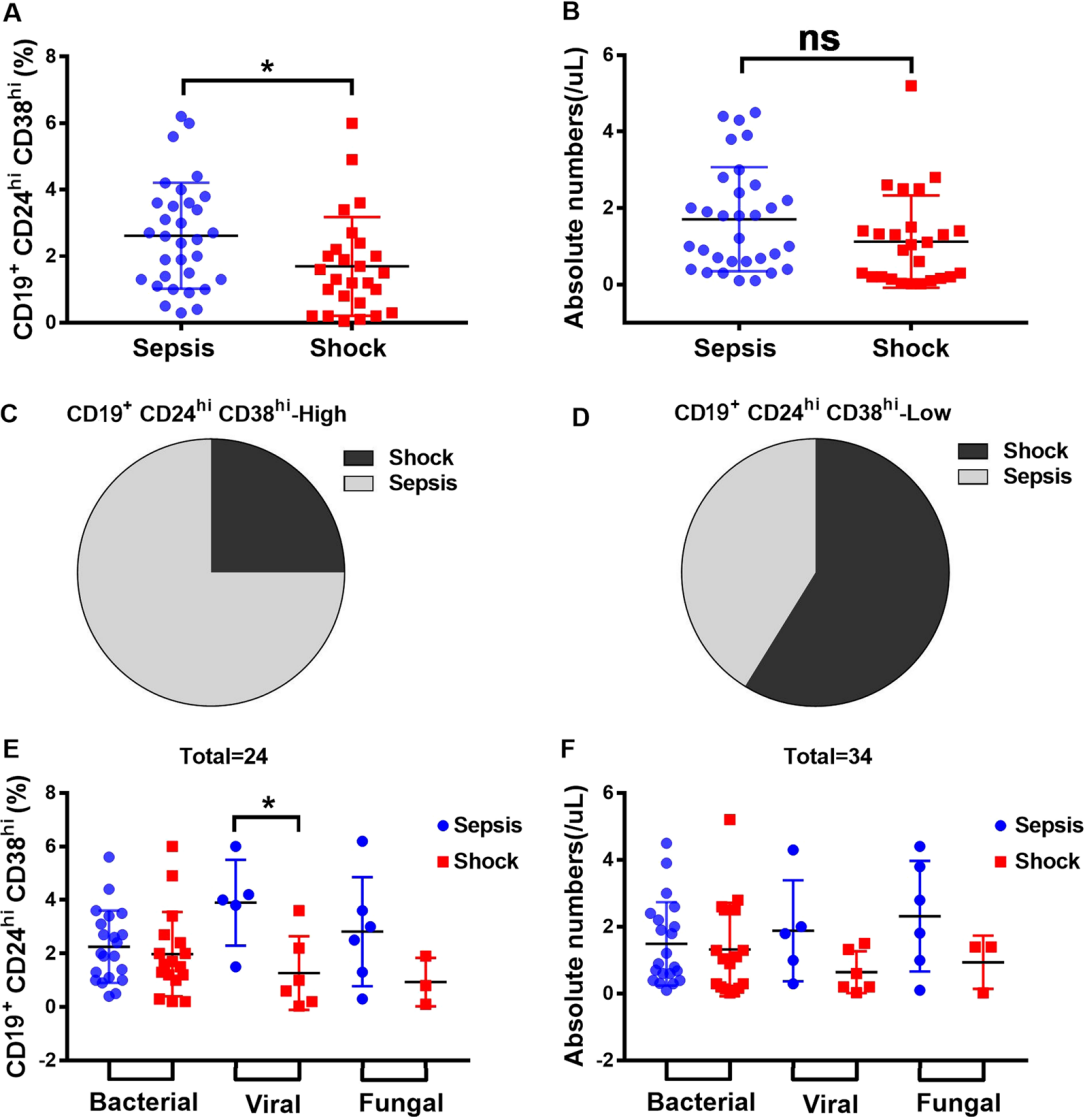
Low frequencies of Bregs were associated with an increased risk of septic shock development in patients with sepsis. **A** The Bregs in sepsis patients that did not develop septic shock versus those that developed septic shock. **B** The absolute count of Bregs in sepsis patients that did not develop septic shock versus those that developed septic shock. **C** Fraction of septic shock patients in Bregs high (%Bregs > 2.204%) patients versus that in Bregs low (%Bregs < 2.204%) patients. **D** Frequency and **F** absolute number of Bregs in sepsis patients versus septic shock patients, subdivided into bacterial, viral and fungal types. Two-tailed t test with unequal variance or Kruskal–Wallis test. *p < 0.05

To investigate the potential role of Bregs in septic shock development in patients with sepsis, we assessed our study cohort for septic shock development (Additional file 2: Table S2). We found that the frequency of Bregs was markedly lower in septic shock group than that in sepsis group, but there was no difference in absolute number of Bregs (Fig. 3A–B). Sepsis patients with high Bregs had reduced risk of developing septic shock. Our study revealed that there was a significantly lower frequency of Bregs in patients who later developed septic shock compared to patients who did not (Fig. 3C, D). When sepsis-related shock was stratified by etiology type, our study revealed that viral sepsis patients who later developed shock had significantly less Bregs frequency, while Bregs counts were not different (Fig. 3E, F).

### IL-10^+^B cells did not match plasma IL-10 in septic patients

**Fig. 4:**
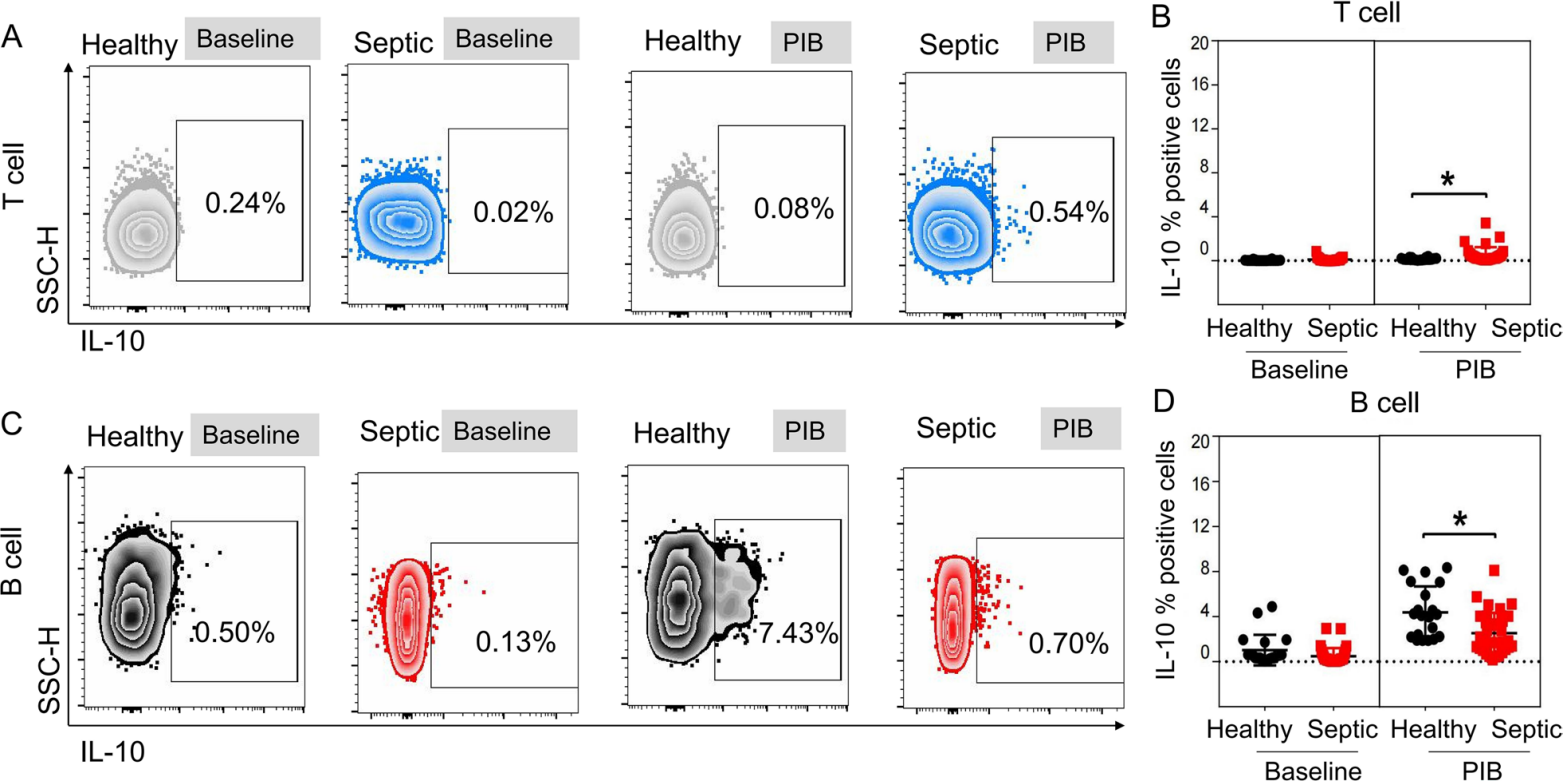
Analysis of IL-10 producing CD4^+^ T cells and CD19^+^ B cells. **A**, **B** Comparison of IL-10^+^ %CD4^+^ T cells between healthy group and sepsis group. **C**, **D** Comparison of IL-10^+^  %CD19^+^ B cells between healthy group and sepsis group. Results are presented as individual values in healthy donors (n = 12; black circles) and septic patients (n = 23; red squares) and medians ± IQR. Two-tailed t-test with unequal variance or Mann–Whitney U test. *p < 0.05

To identify circulating lymphocyte subsets producing IL-10, we detected IL-10 expressed on CD3 and CD19 between septic group and healthy one. Accordingly, we found the proportion of CD4^+^ T cells expressing IL-10 was significantly higher in septic patients than that in healthy group (Fig. 4A, B), while the CD19^+^ B cells expressing IL-10 were lower (Fig. 4C, D). Concentration of plasma IL-10 was significantly higher among septic group when compared to healthy groups (median [IQR] 7.2 [3.4–17.7] vs 1.2 [0.7–2.4], *p* = 0.0211) (Additional file 1: Table S1).

### Bregs frequency was inversely associated with lactate, SOFA and APACHE II and positively correlated with Tregs

**Fig. 5:**
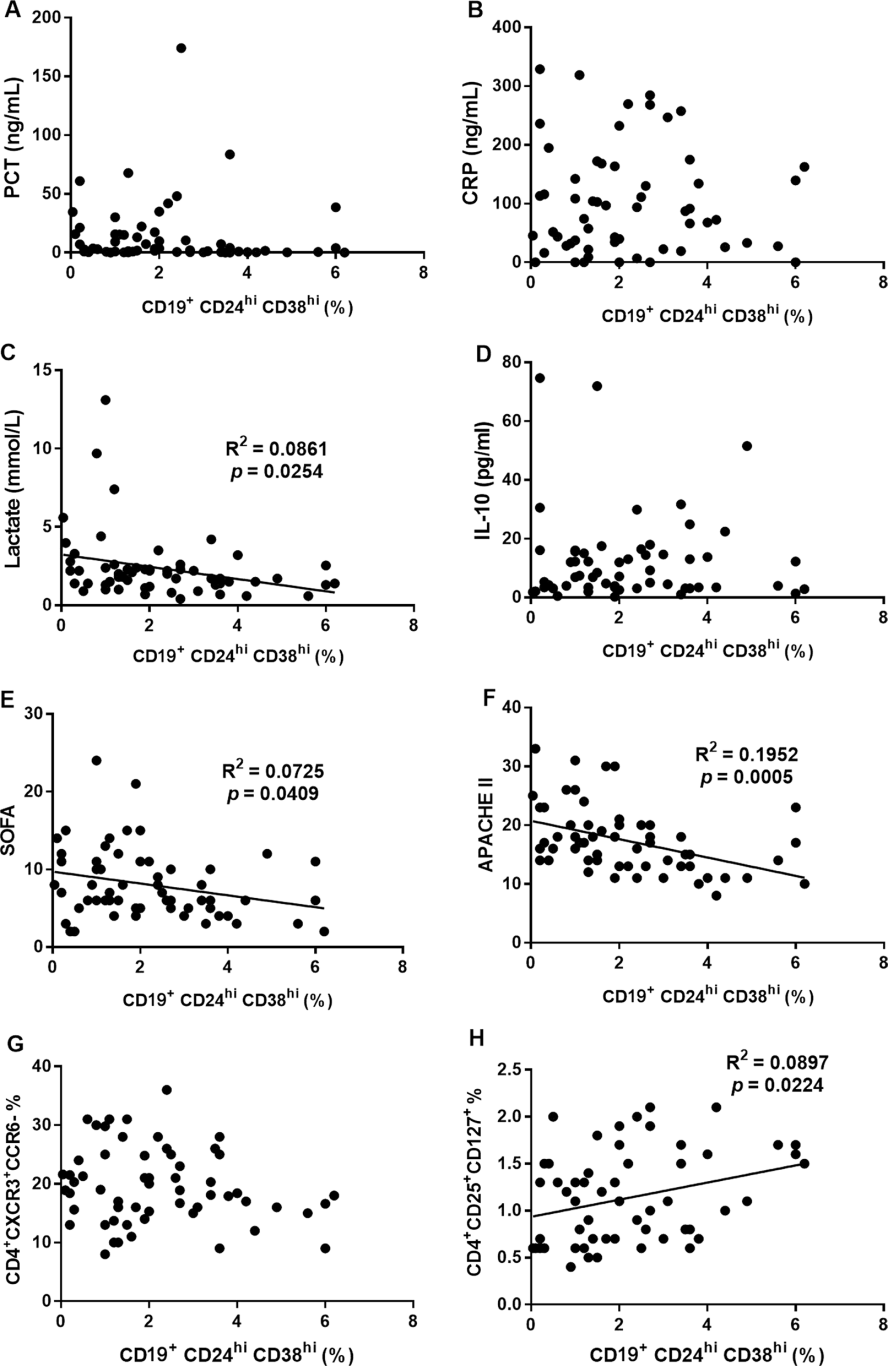
The frequency of Bregs was inversely associated with lactate, SOFA, and APACHE II and positively correlated with Tregs frequency. **A**–**H** The correlations between the frequencies of Bregs and PCT, CRP, lactate, plasma IL-10, SOFA, APACHE II, CD4^+^CXCR3^+^CCR6^−^ T cells, and CD4^+^CD25^+^CD127^+^ T cells in all sepsis patients at day 1 were shown. *p* values represented Pearson’s correlation coefficient

Clinical severity scores such as SOFA, APACHE II and available assays such as blood lactate reflect the severity of septic patients. Here, we found that there were no correlation between Bregs frequency at day 1 and PCT, CRP and plasma IL-10 (Fig. 5A, B, D). Bregs frequency at day 1 was inversely associated with lactate, SOFA, and APACHE II (Fig. 5C, E, F). We examined the correlation between *Bregs* and T cell subsets. There was no correlation between the frequency of *Bregs* and Th1 (Fig. 5G), while *Breg*s were positively correlated with Tregs (Fig. 5H) (R^2^ = 0.0897, *p* = 0.0224).

### Low levels of Bregs were associated with an increased risk of non-survival in septic patients

**Fig. 6:**
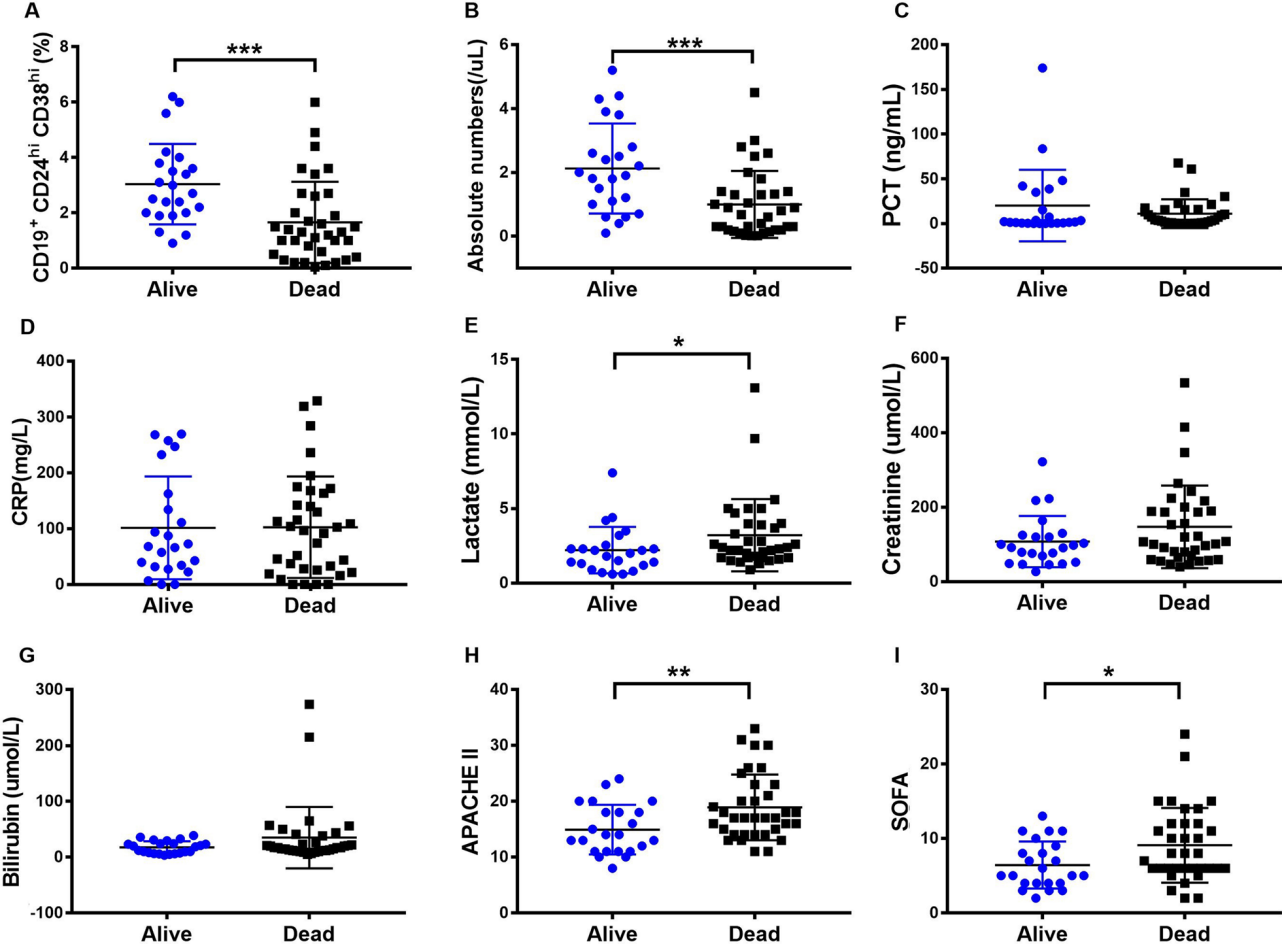
Low levels of Bregs were associated with an increased risk of death in patients with sepsis. **A** Frequency and **B** absolute number of Bregs in sepsis patients were decreased in dead patients. **C** PCT, **D** CRP, **E** lactate, **F** creatinine, **G** bilirubin, **H** APACHE II, and **I** SOFA were compared between alive and dead groups. Two-tailed t-test with unequal variance or Kruskal–Wallis test. *p < 0.05; **p < 0.01; ***p < 0.001

Of the 35 patients, who died during hospitalization, 20 (57.1%) patients had septic shock during their course, meanwhile, 6 out of 23 patients who survived had septic shock (Additional file 3: Table S3, *p* = 0.036). We observed that patients non-survivor had decreased frequency and numbers of Bregs than survivor patients (Fig. 6A–B). Furthermore, lactate, SOFA, and APACHE II, established sepsis severity markers, were elevated in non-survivor group (Fig. 6E, H, I), consistent with a causal association of Bregs changes with non-survivor in septic patients. There were no significant differences in PCT, CRP, creatinine, and bilirubin between *non-survival* group and *survival* group (Fig. 6 C, D, F, G).

### Bregs exerted a good predictive value for 28-day mortality

**Fig. 7:**
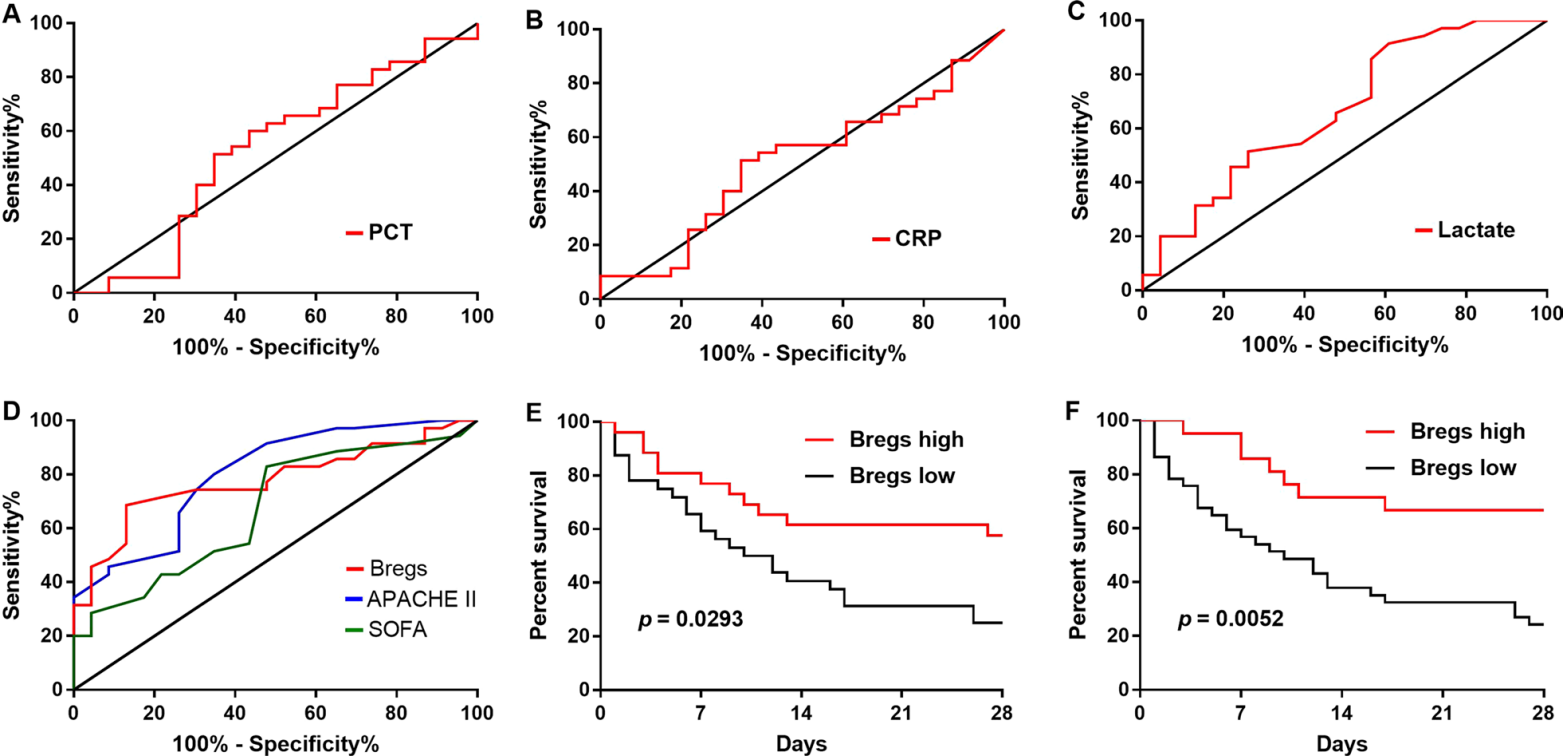
Bregs exerted a good predictive value for 28-day mortality. **A** Survival ROC curve of PCT; **B** Survival ROC curve of CRP; **C** Survival ROC curve of lactate; **D** Survival ROC curve of frequency of Bregs, SOFA score, and APACHE II score; **E** Kaplan–Meier survival curves of frequency of Bregs in Kaplan–Meier plotter cohort; **F** Kaplan–Meier survival curves of absolute number of Bregs in Kaplan–Meier plotter cohort

Mortality predictions were explored for septic patients who died within 28 days. We used the factors mentioned above (Fig. 6) to develop a ROC (receiver operator characteristic curve) for predictive assessment for mortality in septic patients. The area under the ROC curve of PCT, CRP, and lactate were 0.53 (95% CI 0.37–0.69), 0.51 (95% CI 0.36–0.67), 0.67 (95% CI 0.52–0.81) (Fig. 7A–C). The area under the ROC curve of Bregs frequency, SOFA and APACHE II were 0.78 (95% CI 0.66–0.90), 0.67 (95% CI 0.53–0.81), 0.80 (95% CI 0.69–0.92) (Fig. 7D). Moreover, patients with higher frequency and number of Bregs levels (> 2.204%, > 1.444 /µL, respectively) had a significantly better outcome than those with lower levels (< 2.204%, < 1.444 /µL, respectively) (*p* = 0.0293, *p* = 0.0052, respectively) especially in 28-day mortality (Fig. 7E, F).

### Frequency and numbers of Bregs were prediction factors for poor prognosis.

Binary logistic regression analysis (entry method) was performed on the frequency and numbers of Bregs, APACHE II score, and SOFA score, which had statistically significant differences between the survival and the non-survival group, to determine the independent outcome risk factors. The method of screening variables is forward: LR method and the regression coefficient of absolute Bregs value is −0.642, showing a significance level of 0.023 (Table 2, *p* < 0.05), which means that the absolute Bregs number has a significant negative impact on the mortality in septic patients. And the odds ratio (OR value) was 0.526, meaning that when the absolute number of Bregs increased by one unit, the mortality of septic patients decreased by 0.526 times. In a word, the results showed that the numbers of Bregs were independent risk factors affecting the prognosis of elderly septic patients.

**Table 2:** The influence of independent variables on prognosis of patients with sepsis at 28 days

	β	S.E.	Wals	*p*	OR	OR 95% CI	
						Lower	Upper
SOFA score	0.080	0.103	0.608	0.436	1.083	0.886	1.325
Bregs frequency	−0.261	0.251	1.082	0.298	0.770	0.471	1.260
Bregs count	−0.642	0.282	5.169	0.023	0.526	0.303	0.915
APACHE II score	0.118	0.094	1.561	0.211	1.125	0.935	1.354
Stable	−0.553	1.664	0.110	0.740	0.575		

## Discussion

Sepsis is defined as a life-threatening organ dysfunction due to a dysregulated host response following an infection [1]. Sepsis is treatable, and timely implementation of targeted interventions can improve prognosis [22–24]. Because the mechanism of sepsis pathogenesis remains unclear, accurately identifying sepsis patients who are at higher risk of septic shock, while important for public health leaders, healthcare providers, medical personnel, and researchers, remains a great challenge. Recent studies suggest that Bregs have had an essential role in regulating inflammation and autoimmune diseases, very little is known about their involvement in sepsis. Here, we examined the frequency and absolute number of the Bregs population in septic patients at day 1 after admission. We found that septic patients had decreased Bregs frequency and absolute number compared to healthy controls. Moreover, we found a reduced levels of Bregs in sepsis patients who developed septic shock and in non-survival group compared to the survival one. Binary logistic regression analysis identified that Bregs absolute number represented an independent prognostic factor for poor outcome in septic patients.

Bregs can impair immune responses and be linked to increased disease severity during infections. Data on CD19^+^CD24^hi^CD38^hi^ regulatory B cells are conflicting in cohort studies of patients with sepsis. Contrary to our results, Pan X et al. showed that the percentage of CD19^+^CD24^hi^CD38^hi^ regulatory B cells was higher in neonatal patients with sepsis than healthy controls by using flow cytometry [25]. Whereas, the fraction of CD19^+^CD24^hi^CD38^hi^ regulatory B cells on adult patients with sepsis was not altered [26]. Such differences may be attributed to the complexity of sepsis and physiological environments within individuals. In addition to the role in sepsis, a recent study showed Bregs revealed a deficiency proportion of IgM memory and transitional subsets in chronic GVHD [27]. Two other studies confirmed the protective effect of Bregs on mouse cGVHD models [14, 28]. Meanwhile, CD19^+^CD24^hi^CD38^hi^ regulatory B cells are also known to play an important role in the pathogenesis of systemic lupus erythematosus (SLE), because the percentage of CD19^+^CD24^hi^CD38^hi^ regulatory B cells was significantly increased in peripheral blood from patients with SLE [29]. Cui et al. [30] have shown that Bregs significantly decreased in patients with RA.

Moreover, we found a reduced level of Bregs in sepsis patients who developed septic shock. Interestingly, when septic patients were divided into two layers, sepsis and septic shock patients, percent of Bregs was lower in viral septic shock compared to septic patients, indicating the lower the Bregs, the more likely they are to predict viral septic. Peripheral blood CD19^+^CD24^hi^CD38^hi^ regulatory B cells were significantly reduced in patients with severe COVID-19 compared with those with mild disease, which corresponds to the expansion of extrafollicular B cells [31]. We also found Bregs were closely correlated with the progression of sepsis. There was a close correlation between Bregs and progression of sepsis. We observed that there was a positive correlation between the frequency of Bregs and frequency of Tregs in peripheral blood. In particular, we also observed that the frequency of Bregs was inversely associated with artery Lac, SOFA, and APACHE II. It was suggested that the frequency of Bregs had a significant negative correlation with the disease process. Binary logistic regression analysis identified that Bregs absolute number represented an independent prognostic factor for poor outcome in septic patients. Altogether, our results demonstrated that Bregs have been proposed to mediate the anti-inflammatory function and may exert protective effects during sepsis. Bregs have been shown to maintain immune tolerance in humans and mice including secretion of IL-35, TGF-β, IL-10, and through the expression of FasL, GITRL, PD-1, and CD73 [32, 33]. IL-10, a major anti-inflammatory cytokine, suppresses both Th1 and type 2 T helper (Th2) polarization and inhibits antigen presentation and proinflammatory cytokine production by macrophages, lymphocytes, monocytes, dendritic cell and some epithelial cells [34, 35]. Anti-inflammatory response, including the anti-inflammatory cytokine IL-10, IL-LRA, SIL-LR, STNFR-I and STNFR-II, parallel the overproduction of proinflammatory cytokines, may induce an immunosuppressive state in patients with sepsis [36]. We revealed that plasma IL-10 was higher in septic patients than healthy donors, which indicated very early immunosuppression or immunoparalysis in elderly septic patients. Contrary to data from the literature [37], we observed that the ability of B cells secreting IL-10 decreased, which may be due to different stimulation conditions, B cell exhaustion, decreasing numbers and impaired function of B cells. Finally, we observed an increase in IL-10 secreting by CD4^+^ T cells in septic patients, which is consistent with prvious published literature on Tregs in sepsis and septic shock [38, 39]. Previous findings by Roth et al. showed that increased IL-10 concentrations in sepsis may be associated with susceptibility of Th1 to apoptosis, leading to a prevalence of Th2 cells, which is known for their IL-10 production. CD14^+^ monocytes, not CD4^+^ T cells nor multipotent adult progenitor cells, are resposible for IL-10 production [40]. In addition, B cells from acute COVID-19 patients showed increased IL-6 and decreased IL-10 production compared with healthy B cells. Age-related declines in the number and function of Bregs may contribute to "inflammation" or the “chronic inflammation” observed with ageing [41]. Age‐associated reductions in Bregs frequency, as well as impaired IL‐10 production, were observed in healthy older donors (> 60 years) compared to healthy younger donors (20‐40 years old) [41]. Patients that succumbed to shock might be due to B cells exhibited a decrease in IL-10 secretion, indicating that both anti-inflammatory cytokines, inhibition of B cells in the initial stage of sepsis in elderly patients, and exogenous IL-10 had protective effects in sepsis.

APACHE II score is a classical standard to evaluate the severity of the disease in critically ill patients, and patients can be classified according to the severity of the disease. Studies have shown that APACHE II score is negatively correlated with ICU length of stay, and positively correlated with mortality [42]. SOFA score is also a simple and effective method to evaluate organ dysfunction/failure in critically ill patients. High SOFA score is associated with high mortality [42]. Our study demonstrated that APACHE II score, SOFA score, lactate, and Bregs differed significantly between dead and alive patients. Moreover, percentage of Bregs were negatively correlated with severity scores, and significantly correlated with non-survival of septic patients. All this data indicates that changes in Bregs may predict the prognosis in septic patients.

## Conclusions

Overall, our results indicate that patients with sepsis had a lower frequency and number of Bregs, which seemed to be closely associated with disease progression, predicting a shorter overall survival and poor prognosis. Circulating Bregs numbers may be used as a reliable supplementary resource in the determination of sepsis severity. A longitudinal study of Bregs activity in sepsis patients should be explored as a test for disease progression/resolution. It is noteworthy that Bregs provides a novel insight into the dynamics underlying sepsis progression. All these data suggest Bregs have a potential immunosuppressant function in sepsis.

## Supplementary Information

**Additional file 1**. **Table S1**. Clinical and laboratory data in septic patients and healthy donors.

**Additional file 2**. **Table S2**. Clinical and laboratory data between septic and septic shock patients.

**Additional file 3**. **Table S3**. Clinical and laboratory data between survivors and non-survivors of sepsis.

**Additional file 4**. All original data generated or analyzed in the study.

## Data Availability

All original data generated or analyzed in the study are included in this article/supplementary materials (Additional file 4). Further inquiries can be directed to the corresponding author.

## Abbreviations

ANOVA: One-way analysis of variance
APACHE II: Acute physiology and chronic health evaluation II
Bregs: Regulatory B cells
cGVHD: Chronic graft-versus-host disease
CRP: C-reactive protein
HBV: Hepatitis B virus
ICU: Intensive care unit
IL-10: Interleukin-10
Lac: Lactate
mNGS: Metagenomic next generation sequencing
PBMCs: Peripheral blood mononuclear cells
PCT: Procalcitonin
ROC: Receiver operator characteristic curve
Th1: Type 1 T helper
Th2: Type 2 T helper
Tregs: Regulatory T cells
SOFA: Sequential organ failure assessment
WBC: White blood cell
