# Supplementary material for: CD19+CD24hiCD38hi regulatory B cells deficiency revealed severity and poor prognosis in patients with sepsis
Source: BMC Immunol. 2022 Nov 10;23:54. doi: 10.1186/s12865-022-00528-x (PMC9648441; doi:10.1186/s12865-022-00528-x)
Supplement: Supplementary file 1 — Additional file 1. Table S1. Clinical and laboratory data in septic patients and healthy donors. [file 12865_2022_528_MOESM1_ESM.doc]

**Supplemental Table 1** Clinical and laboratory data in septic patients and healthy donors

|  | Healthy | Septic | t | *p* |
| --- | --- | --- | --- | --- |
| Age (year) | 75.2 ± 7.0 | 78.6 ± 8.1 | 1.832 | 0.0707 |
| Gender (male/female), n | 10 / 12 | 38 / 20 | 0.1667 | 0.1281 |
| ICU days | / | 7.5 (1-11) |  |  |
| Vasoactive agents use, n (%) | / | 27 (46.6%) |  |  |
| SOFA score | / | 8.0 ± 4.0 |  |  |
| APACHEII score | 8.5 ± 1.2 | 17.3 ± 3.2 | 7.302 | <0.0001 |
| WBC ( /μL) | 5.9 ± 0.8 | 16.8 ± 2.2 | 6.177 | <0.0001 |
| B cell ( /μL) | 87.5 ± 10.0 | 58.0 ± 5.7 | 2.636 | 0.0101 |
| Platelets ( /μL) | 182.6 ± 9.8 | 143.2 ± 13.7 | 1.716 | 0.0901 |
| Bilirubin (μmol/L) | 8.0 ± 0.6 | 28.8 ± 5.7 | 2.143 | 0.0352 |
| Creatinine (μmol/L) | 74.6 ± 6.8 | 131.5 ± 12.9 | 2.711 | 0.0083 |
| PCT (ng/mL) | 0.09 ± 0.03 | 14.40 ± 6.00 | 2.399 | 0.0188 |
| CRP (mg/L) | 3.2 ± 0.2 | 102.2 ± 12.8 | 5.098 | <0.0001 |
| Lactate (mmol/L) | 0.4 ± 0.1 | 2.8 ± 0.4 | 5.193 | <0.0001 |
| IL-10 (pg/mL, IQR) | 1.2 (0.7, 2.4) | 7.2 (3.4, 17.7) | 2.373 | 0.0211 |
| Ratio of Bregs to Th1 cells | 0.37 ± 0.05 | 0.13 ± 0.02 | 5.599 | <0.0001 |
| Ratio of Bregs to Treg cells | 1.61 ± 0.26 | 2.12 ± 0.20 | 1.384 | 0.1702 |
