# Supplementary material for: CD19+CD24hiCD38hi regulatory B cells deficiency revealed severity and poor prognosis in patients with sepsis
Source: BMC Immunol. 2022 Nov 10;23:54. doi: 10.1186/s12865-022-00528-x (PMC9648441; doi:10.1186/s12865-022-00528-x)
Supplement: Supplementary file 2 — Additional file 2. Table S2. Clinical and laboratory data between septic and septic shock patients. [file 12865_2022_528_MOESM2_ESM.doc]

**Supplemental Table 2** Clinical and laboratory data between septic and septic shock patients

|  | Septic | Septic shock | *p* |
| --- | --- | --- | --- |
| Age (year) | 79.8 ± 8.4 | 80.0 ± 9.6 | 0.9157 |
| Gender (male/female), n | 21 / 11 | 17 / 9 | 0.9999 |
| ICU days | 8 (1, 9) | 6 (2, 10) | 0.6453 |
| Vasoactive agents use, n (%) | 5(21.7) | 22 (95.6) | <0.0001 |
| WBC ( /μL) | 16.7 ± 2.6 | 16.8 ± 2.7 | 0.9936 |
| Platelets( /μL) | 172.0 ± 19.7 | 107.7 ± 16.5 | 0.0179 |
| Bilirubin (μmol/L) | 16.7 ± 1.7 | 42.3 ± 12.2 | 0.0262 |
| Creatinine (μmol/L) | 113.7 ± 17.8 | 153.5 ± 19.1 | 0.1238 |
| PCT (ng/mL) | 8.7 ± 5.4 | 21.8 ± 4.6 | 0.0017 |
| CRP (mg/L) | 95.9 ± 15.3 | 109.9 ± 19.0 | 0.5643 |
| Lactate (mmol/L) | 2.2 ± 0.2 | 3.5 ± 0.5 | 0.0206 |
| Bregs ( %) | 2.6 ± 0.3 | 1.7 ± 0.3 | 0.0273 |
| Bregs ( /μL) | 1.7± 0.2 | 1.1± 0.2 | 0.0920 |
