# Supplementary material for: CD19+CD24hiCD38hi regulatory B cells deficiency revealed severity and poor prognosis in patients with sepsis
Source: BMC Immunol. 2022 Nov 10;23:54. doi: 10.1186/s12865-022-00528-x (PMC9648441; doi:10.1186/s12865-022-00528-x)
Supplement: Supplementary file 3 — Additional file 3. Table S3. Clinical and laboratory data between survivors and non-survivors of sepsis. [file 12865_2022_528_MOESM3_ESM.doc]

**Supplemental table 3** Clinical and laboratory data between survivors and non-survivors of sepsis group

| Variables | Survivors | Non-survivors | *p* |
| --- | --- | --- | --- |
| Cases, n | 23 | 35 |  |
| Age(year) | 80.1 ± 6.9 | 79.7 ± 7.1 | 0.869 |
| Gender (male/female) | 14 / 9 | 24 / 11 | 0.583 |
| Septic shock, n (%) | 6 (26.1%) | 20 (57.1%) | 0.036 |
| PCT (ng/mL) | 10.9 ± 2.7 | 19.9 ± 8.3 | 0.236 |
| CRP(mg/L) | 102.6 ± 15.4 | 101.5 ± 19.2 | 0.962 |
| Lactate (mmol/L) | 2.2 ± 0.3 | 3.2 ± 0.4 | 0.031 |
| Bregs ( %) | 3.0 ± 0.3 | 1.7 ± 0.3 | 0.001 |
| Bregs ( /μL) | 2.1 ± 0.3 | 1.0 ± 0.2 | 0.001 |
